# Persistence of *Salmonella* Typhimurium in Well Waters from a Rural Area of Changchun City, China

**DOI:** 10.3390/ijerph15061090

**Published:** 2018-05-28

**Authors:** Jiahang Li, Meiyue Ding, Ziming Han, Jincai Ma

**Affiliations:** 1 Key Laboratory of Ground Water Resource and Environment, Ministry of Education, Jilin University, Changchun 130021, China; lijiahang96@163.com (J.L.); dingmy1013@163.com (M.D.); hanzm2514@mails.jlu.edu.cn (Z.H.); 2 College of Environmental and Resources, Jilin University, Changchun 130021, China

**Keywords:** *Salmonella* Typhimurium, persistence, well water, NH_4_^+^–N, pH

## Abstract

*Salmonella*-contaminated well water could cause major infection outbreaks worldwide, thus, it is crucial to understand their persistence in those waters. In this study, we investigated the persistence of *Salmonella enterica* serovar Typhimurium in 15 well waters from a rural area of Changchun City, China. Results illustrated that the time to reach detection limit (*ttd*), first decimal reduction time (*δ*), and the shape parameter (*p*) ranged from 15 to 80 days, from 5.6 to 66.9 days, and from 0.6 to 6.6, respectively. Principal component analysis showed that *ttd*s of *S. Typhimurium* were positively correlated with total organic carbon, pH, NH_4_^+^–N, and total phosphate. Multiple stepwise regression analysis revealed that *ttd*s could be best predicted by NH_4_^+^–N and pH. Canonical correspondence analysis and variation partition analysis revealed that NH_4_^+^–N and pH, and the rest of the water parameters, could explain 27.60% and 28.15% of overall variation of the survival behavior, respectively. In addition, *ttd*s were found to be correlated (*p* < 0.01) with *δ* and *p*. Our results showed that the longer survival (>2.5 months) *S. Typhimurium* could constitute an increased health risk to the local communities, and provided insights into the close linkage between well water quality and survival of *S. Typhimurium*.

## 1. Introduction

*Salmonella* has been recognized as one of the worldwide human zoonotic pathogens. When infected, patients can display typical symptoms including typhoid, fever, and diarrhea, which could be life-threatening among the young, the elderly, and immune-deficient individuals [1]. Consumption of pork, beef, poultry meat, and eggs is believed to be the major cause of *Salmonella* infection [2,3]. Accumulated evidence has shown that *Salmonella* is one of the major threats for people’s health. In the United States alone, approximately 1.4 million cases of salmonellosis were reported annually [4]. In China, infection outbreaks caused by *Salmonella* accounted for about 40% of the total bacterial infection annually [5]. A foodborne *S*. Typhimurium infection outbreak was reported to occur in Guangxi, China, and this outbreak led to 108 infections [6]. Drinking water, either poorly chlorinated or bacterial contaminated, was the major vector for *Salmonella* infection. A large community-wide outbreak that occurred in Alamosa, Colorado, was believed to be caused by unchlorinated municipal drinking water contaminated by *S*. Typhimurium, and this outbreak resulted in 124 confirmed cases, 20 hospitalizations, and 1 death [7]. In the Zhejiang Province of China, 26 cases were reported in an outbreak that was caused by well water contaminated by *Salmonella* [8].

The major reservoir of *Salmonella* is poultry litter, and manure of other animals, including pigs, cattle, as well as some wild animals that are also being recognized as potential sources of this pathogen [9]. When *Salmonella*-laden manure is applied into farms as organic fertilizer, the pathogen may be mobilized and transported to other places via surface runoff and wind, and thus redistributed in water, air, soil, and even in subsurface environments, e.g., groundwater [10]. The persistence and regrowth of pathogen in such environments could increase their opportunity to enter into the food chain and jeopardize human health [11]. Therefore, it is crucial to investigate the persistence of this pathogen in those environments in order to prevent potential health risks.

Groundwater supplies were thought to be free of pathogenic microbes, due to the natural filtering ability of the subsurface environment and to the long distance for pathogens to reach the groundwater. However, mismanagement of animal waste disposal and shallow wells may lead to microbial contamination [12]. Domestic animals and farms nearby might contribute to fecal contamination of private wells, and result in outbreaks of diseases [13]. Similarly, a Canadian study revealed that individual exposure to contaminated private well water in a rural area may lead to an increased risk of acute gastrointestinal illness [14]. In southern Changchun city, China, private well water is a unique drinking water source, as dwellers have no access to municipal water supply. As a result, the untreated well water might increase the risk of bacterial infection.

In this study, we collected 15 well water samples from small villages in southern Changchun city, China, and determined physical and chemical properties of those samples. The objectives of our study were to (1) understand the survival of *S*. Typhimurium in 15 well water samples, and (2) identify the environmental factors affecting the survival of *S*. Typhimurium.

## 2. Material and Methods

### 2.1. Study Site

The well water samples were collected from a rural area, which was about 20 km south of Changchun city, China. Local people there use well water as their unique source of drinking water, since tap water is unavailable. Well water samples were collected from 15 small villages, and the well location, depth, and water temperature were recorded (Table 1). The well shared by the maximum number of population in each village was our targeted sampling well.

### 2.2. Well Water Sampling and Characterization

A total of 15 wells were sampled, among them 2 were covered open wells, and the rest were tube wells. For open well sampling, grab sampling technique was used; for tube well sampling, water was sampled only after the existing water in the tube was completely replaced by a large volume of fresh water [15]. All samples were collected in the morning, when maximum water usage was reached for the local community [15]. Duplicate samples per well were collected. Samples were saved in clean and sterile polypropylene containers and transported to the laboratory on ice, and their characterization was done within 6 h of collection.

Total organic carbon (TOC) and total nitrogen (TN) in water samples were determined by TOC (TOC-L, Shimazu, Japan), NH_4_^+^–N was determined by Nessler’s reagent spectrophotometry method, NO_3_^−^–N was quantified by double wavelength ultraviolet spectroscopy method. Total phosphorus (TP) was quantified by potassium persulfate oxidation coupled with ammonium phosphomolybdate colorimetry. Total soluble iron (Fe) was examined by ascorbic acid reduction–phenanthroline spectrophotometric analysis method. Total plate counting (TPC) was conducted to numerate bacteria well water samples (data not shown due to the lack of significant correlation with *ttd*s). The results of well water characterization were shown in Table 1.

### 2.3. S. Typhimurium Strain

*Salmonella enterica subsp. enteric* serovar Typhimurium (ATCC 14028) was used as inoculum strain. In order to facilitate plate counting, wild type *S. Typhimurium* was tagged with nalidixic acid and rifampicin resistance marker via spontaneous mutation protocol [16]. In brief, the rifampicin resistant strain was screened by plating wild type strain on LB (Luria-Bertani) agar supplemented with rifampicin (100 mg/L), and then the rifampicin- and nalidixic acid-resistant strain was screened on LB agar supplemented with nalidixic acid (20 mg/L). The apparent growth rate of the tagged strain was identical to that of wild type (data not shown).

### 2.4. Growth and Numeration of S. Typhimurium

Cells of *S. Typhimurium* were streaked onto the LB agar media and incubated under 37 °C overnight. Single colonies were inoculated from the media, and streaked onto the LB agar with nalidixic acid and rifampicin. Single colonies from this selective media were inoculated in 100 mL LB broth in 37 °C for 16 h. The broth cultures were separated by centrifugation (8000× *g* at 4 °C), washed with 0.9% NaCl three times to remove the residues of nutrients from LB broth and then resuspended in sterile deionized water. Stationary phase cells were used, and they were spiked into the well water samples to a final cell density of 10^4^ colony forming units (cfu)/mL [17,18]. The well water was put into a 5 mL carbon free glass tube, kept in dark under room temperature (21 ± 1 °C). The samples were mixed two times per day to simulate regular drinking water storage conditions in the sampling area. All samples were prepared in triplicate. Well waters were sampled at different time intervals (1 to 7 days), then subjected to 10-fold serial dilutions, and plated onto antibiotic supplemented LB agar. The antibiotics, rifampicin (Rif) and nalidixic acid (Nal) were added into the LB agar media at 100 mg/L and 20 mg/L, respectively. Negative control experiments, including well water and filtered (0.45 μm) well water, were conducted to investigate the potential contamination of water samples during the course of the experiments.

### 2.5. Survival Data Modeling

Survival of *S. Typhimurium* was analyzed by fitting the experimental data to the Weibull survival model using GInaFiT version 1.5 developed by Dr. Annemie Geeraerd [19,20]. This model was based on the hypothesis that the strain follows a Weibull distribution. The size of the surviving population can be calculated using the following equation:

(1) $$\log\left(N_{t}\right)=\log\left(N_{0}\right)-{\left(\frac{t}{\delta }\right)}^{p},$$

where *N* is number of survivors, $N_{0}$ is inoculum size, *t* is time (days) post-inoculation, *δ* is scale parameter representing the time needed for the first decimal reduction, and *p* is non-unit shape parameter. When *p* > 1, a convex curve was observed, when *p* < 1, a concave curve was observed, and when *p* = 1, a linear curve was observed. The parameter of time (days) needed to reach detection limit, *ttd*, can be calculated by using GInaFiT to fit the experimental survival data. The detection limit was 10 cfu/mL.

### 2.6. Statistical Analysis

Principal component analysis (PCA) of various well water physical and chemical characteristics, including pH, EC (dS/m), TP (μg/L), TOC (mg/L), TN (mg/L), NH_4_^+^–N (mg/L), NO_3_^−^–N (mg/L), total soluble iron (mg/L), and *ttd* (day), was conducted using PC-ORD v5.0 (MjM Software, Gleneden Beach, OR, USA). Linear regression analysis on *ttd* with *δ* and *p*, and cluster analysis (Ward’s method based on squared Euclidean distance) of well water samples based on *ttd* were performed by SPSS 19 (IBM, Armonk, NY, USA). Stepwise multiple regression analysis was also conducted to establish the correlation between *ttd* and the water characteristics by using SPSS 19. Canonical correspondence analysis (CCA) and variation partition analysis (VPA) were performed using R v.3.3.2 (R Foundation for Statistical Computing, Vienna, Austria) with the Vegan package, and log_2_(X + 1) transformed variables were used in CCA and VPA. Path analysis was conducted by R v.3.3.2 with the Agricolae package.

## 3. Results

### 3.1. Well Water Characterization

The results of well water sample characterization (Table 1) illustrated that each well had a depth less than or equal to 60 m, and the water temperature was between 5.4 and 8.9 °C. The pH of all well water samples fell into a small range of values between 6.38 and 7.40. According to Chinese Standards for Drinking Water Quality (GB5749-2006), the NH_4_^+^–N concentration of sample 9 was larger than the maximal level (0.5 mg/L), and the NO_3_^−^–N concentrations of sample 3, 10, 12, and 13 were greater than the upper limit (20 mg/L), indicating potential contamination by local agricultural activities, e.g., inorganic fertilizer application and animal farms. The salinity, as indicated by electrical conductivity (EC), was relatively larger for sample 3, 9, and 13, than the rest of the samples.

### 3.2. Survival Profiles of S. Typhimurium in Well Waters

**Figure 1:**
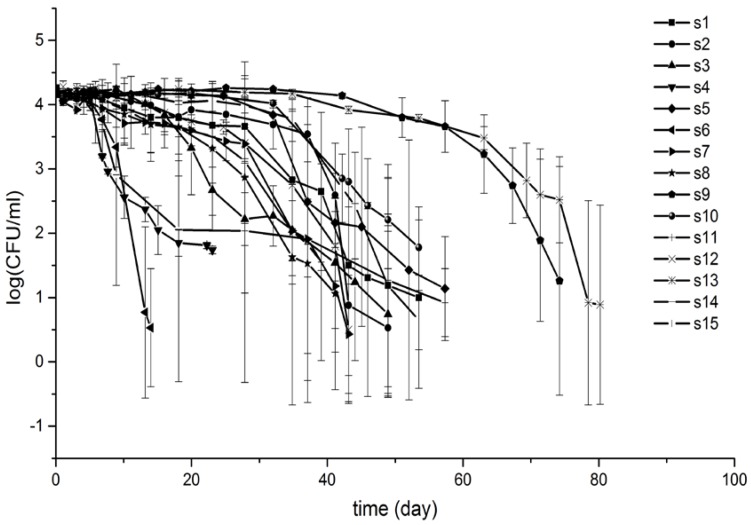
Survival curves of *S. Typhimurium* in well water samples.

The survival profiles of *S. Typhimurium* in each of the well water samples are shown in Figure 1. There was a significant difference in *ttd* among each water sample. Sample 4 and 6 had *ttd*s less than 20 days, while Sample 9 and 13 persisted for more than 70 days, while in other well water samples, *ttd*s of *S. Typhimurium* were between 30 and 50 days. Survival profiles of s4 and s14 showed concave curves indicating *p* < 1, while the survival profiles of the rest of the samples displayed convex curves. The first decimal times (*δ*) for s4, s6, and 14 were less than 10 days, while those for s9 and s13 were more than 60 days, and *δ* for the rest of samples were between 18 and 40 days.

### 3.3. Cluster Analysis

**Figure 2:**
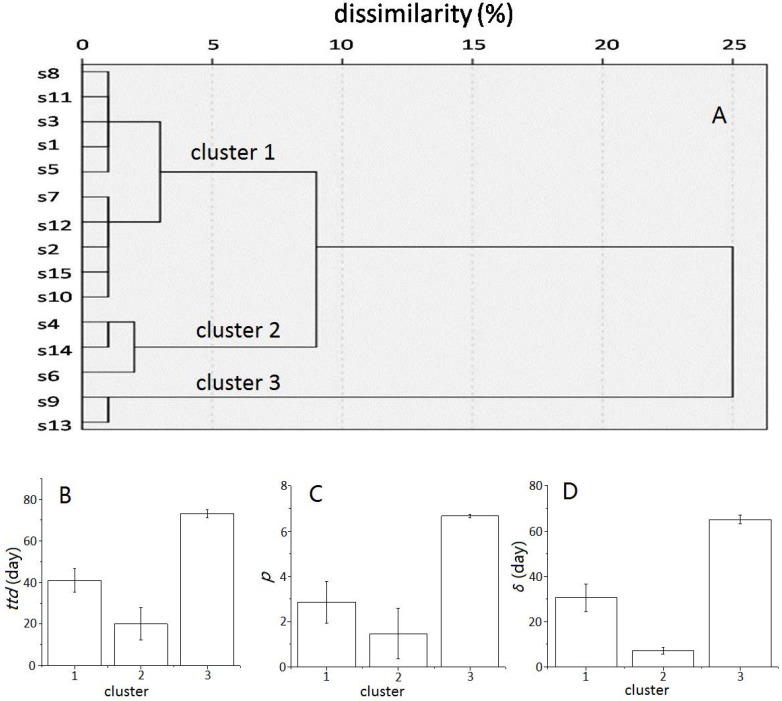
Cluster analysis (**A**) of well water samples based on the survival parameters, including time to detection limit (*ttd*), shape parameter (*p*), and the first decimal reduction time (*δ*), and comparison of mean values of *ttd* (**B**), *p* (**C**), and *δ* (**D**) in each cluster.

Hierarchical cluster analysis yielded three clusters (Figure 2A). Cluster 2 included three samples (s4, s6, s14), cluster 3 included two samples (s9, s13), and the rest of samples belonged to cluster 1. The mean values of *ttd*, *p*, and *δ* in each cluster (Figure 2B–D) showed that the mean *ttd* in cluster 3 (73.1 days) was significantly longer than those in cluster 1 and 2 (41.1 and 20.9 days, respectively). The average of *p* in cluster 2 (1.5) was shorter than those in cluster 1 and 3 (2.9 and 6.7, respectively). Similarly, samples in cluster 3 had a greater mean value of *δ* (65.0) compared with the samples in cluster 1 and 2 (30.6 and 7.1, respectively).

### 3.4. Principal Component Analysis of Survival Data and Water Properties

**Figure 3:**
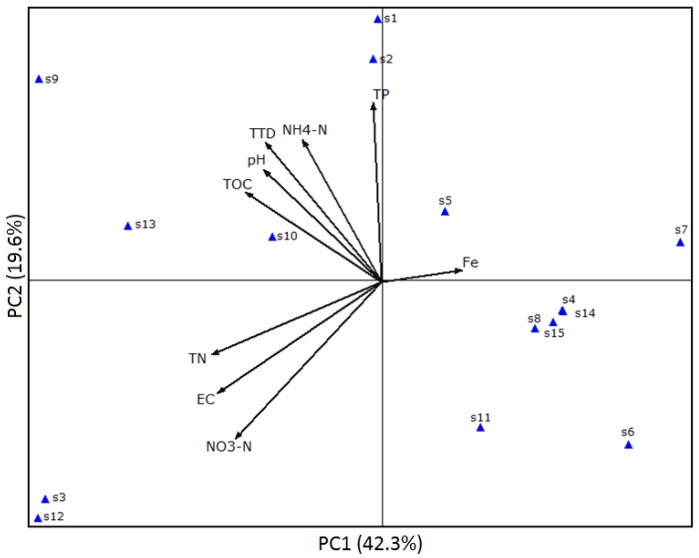
Principal component analysis of survival time (*ttd*) and water physicochemical properties. EC, electrical conductivity; TOC, total organic carbon; TN, total soluble nitrogen; TP, total soluble phosphorus; Fe, total soluble iron.

In this study, many physicochemical factors had influence on the survival of *S. Typhimurium*. PCA was used to determine the effects of water physical and chemical parameters on the survival of *S. Typhimurium*. PCA results in Figure 3 showed that the first two PCs accounted for 61.9% of the total variance, with PC1 accounting for 42.3%, and PC2 accounting for 19.6%. According to PC1, NH_4_^+^–N, pH and TOC exhibited positive scores apparently, indicating that these factors may have positive effects on the survival of *S. Typhimurium*.

### 3.5. Multiple Stepwise Regression Analysis

In order to quantify the results of principal component analysis, multiple stepwise regression analysis (Table 2) was conducted, and the results showed that NH_4_^+^–N and pH were the best factors predicting the survival time of *S. Typhimurium*, with NH_4_^+^–N (*p* < 0.05) and pH (*p* < 0.1) having positive effects on *ttd*.

### 3.6. Path Analysis

**Figure 4:**
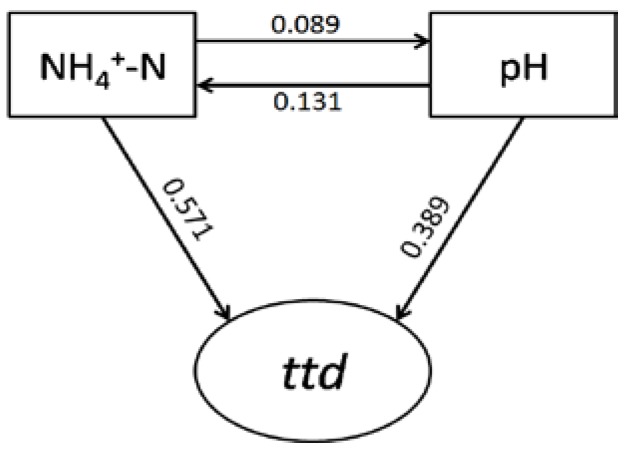
Path analysis of survival time (*ttd*) and water physicochemical properties (NH_4_^+^–N, pH).

Path analysis (Figure 4) indicated that NH_4_^+^–N and pH showed direct effects on *ttd*s. Both factors also displayed indirect effects on *ttd*s, with NH_4_^+^–N influencing *ttd*s via affecting pH, and pH influencing *ttd*s via affecting NH_4_^+^–N.

### 3.7. Canonical Correspondence Analysis and Variation Partition Analysis

**Figure 5:**
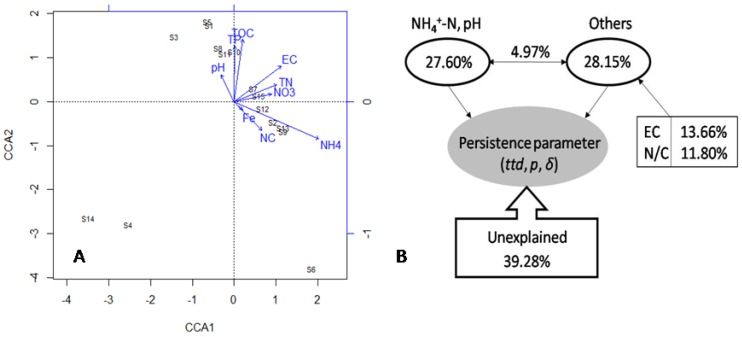
Canonical correspondence analysis of the survival parameters, including time to detection limit (*ttd*), shape parameter (*p*), and the first decimal reduction time (*δ*), and water physicochemical properties. EC, electrical conductivity; TOC, total organic carbon; TN, total soluble nitrogen; TP, total soluble phosphorus; Fe, total soluble iron; NC, the ratio of total nitrogen to total carbon (**A**). Variation partition analysis of the effects of NH_4_^+^–N, pH and other water physicochemical properties on the survival parameters (*ttd*, *p*, and *δ*) of *S. Typhimurium* (**B**).

The CCA plots (Figure 5A) indicated that TOC, EC, TP, NH_4_^+^–N, NO_3_^−^–N, N/C, Fe, pH, and TN influenced the survival time, of which NH_4_^+^–N was more correlated with the survival of *S. Typhimurium*. According to VPA in Figure 5B, NH_4_^+^–N and pH explained 27.60% while other properties explained 28.15% of the variation of the survival data (*ttd*, *p* and *δ*), leaving 39.28% unexplained.

### 3.8. Linear Correlation of ttd with δ and p

**Figure 6:**
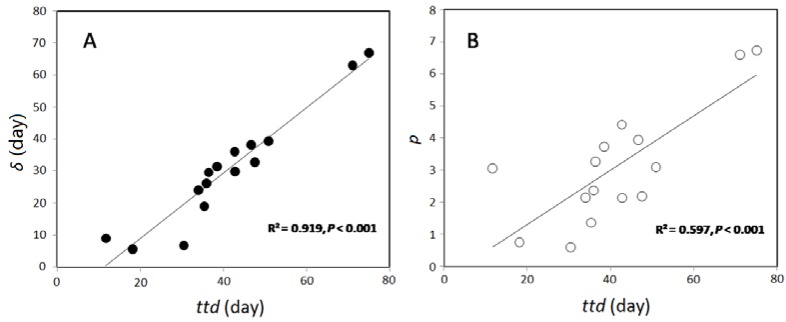
Correlation of survival time (*ttd*) with (**A**) first decimal reduction time (*δ*) and (**B**) the shape parameter (*p*).

Linear correlation in Figure 6 revealed that *ttd*s were significantly correlated (*p* < 0.001) with the first decimal reduction time, *δ*, and the shape parameter, *p*.

## 4. Discussion

Analysis of our study illustrated that 1 and 4 of well water samples exceeded upper limits of the Chinese Standards for Drinking Water Quality (GB5749-2006) in terms of NH_4_^+^–N and NO_3_^−^–N, respectively. In our sampling area, domestic animals and farms were sporadically distributed. Animals such as cows and pigs may be the source of fecal contamination to well waters. Also, well water might get contaminated by the overuse of fertilizer in local agricultural farms. According to a previous survey, ammonia in environmental waters can be a good indicator for fecal contamination, and a high level of NH_4_^+^–N was frequently linked to fecal contamination [21]. On the other hand, a previous study showed that the high level of nitrate was related to cattle farming [22], and the depth of shallow wells was often associated with higher level of nitrate concentrations [23]. However, our data showed that there was no strong relationship between the depths of these wells and NO_3_^−^–N concentrations, suggesting the contribution of the other potential sources to nitrate load in well waters. The NO_3_^−^–N based inorganic nitrogen fertilizer applied into the local farms around the sampling areas might be the major contributor [24].

In our study, the survival of *S. Typhimurium* displayed various survival profiles in well water samples under lab-simulated storage conditions. Our study showed that the longest survival was 75.1 days, and the shortest was 11.7 days. The average *ttd* was 41.1 days, which was comparable to the survival time, 45 days, of a *Salmonella* strain inoculated into untreated river water [25]. Our results were also consistent with another report showing that *Salmonella* could survive for more than 63 days in water mixed with manure [26]. It was also revealed that our *ttd*s in well water samples were highly in line with those observed in soils [27,28] and in sediment [29]. It is worth to mention that the *ttd*s in current study were comparable with those of other human pathogens, e.g., *E. coli* O157:H7 in water samples [30].

Cluster analysis showed that the longest survival of *S. Typhimurium* was observed in cluster 3 samples (s9 and s13), with *ttd*s being 71.1 and 75.1 days, respectively (Figure 4), and such a long survival time was coincident with high levels of NH_4_^+^–N, with the corresponding concentrations being 0.696 mg/L and 0.422 mg/L, respectively. A previous survey indicated that the survival of *S. Typhimurium* in water amended with manure was more than 60 days [26]. Such higher persistence could be due to the existence of manure containing additional nutrients, e.g., NH_4_^+^–N, which might lead to longer survival times.

PCA results (Figure 3) indicated that *ttd*s were positively related with several physicochemical factors, such as NH_4_^+^–N, pH, and TOC, which displayed the same trend observed in cluster analysis. Path analysis (Figure 6) was applied to probe the key factors (NH_4_^+^–N, pH) controlling the survival of human pathogen in environmental samples [28,31]. Both cluster analysis and PCA results indicated that NH_4_^+^–N and pH played a significant role in the survival of *S. Typhimurium*, and both of the two factors were positively correlated with the *ttd*s.

Results of multiple stepwise regression analysis (Table 2) showed both NH_4_^+^–N and pH displayed positive correlations with the *ttd*s. Results of CCA, VPA (Figure 5B), and multiple stepwise regression analysis showed a common trend, that NH_4_^+^–N and pH were the major well water parameters controlling the overall survival behavior of *S. Typhimurium*. NH_4_^+^–N, pH, and other measured well water properties explained 55.75% of the overall variation of survival behavior of *S. Typhimurium*, leaving 39.28% unexplained. The unmeasured factors, such as dissolved oxygen, redox potential, trace element concentrations, and microbial community composition and structure of well waters could contribute to the unexplained portion of the overall variation of survival behavior of *S. Typhimurium*.

Our results showed that the concentration of NH_4_^+^–N was a significant factor influencing the survival of *S. Typhimurium*. According to previous studies, bacteria prefer to use NH_4_^+^–N as their nitrogen source [32,33,34], although some bacteria are capable of assimilate NO_3_^−^–N [35]. The reasons might lie in the fact that microbes consume less energy when using NH_4_^+^–N as their nutrient source [33]. When NH_4_^+^–N remains insufficient, bacteria may use NO_3_^−^–N as their nitrogen source. During this period of time, bacteria would consume more energy, which might increase their energy load for NO_3_^−^–N assimilation. Therefore, a relatively higher level of NH_4_^+^–N may favor the persistence of *S. Typhimurium* in well waters tested in the current study.

We observed that pH was positively correlated with the persistence of *S. Typhimurium* in well water samples (Table 2). Water pH may indirectly affect the *S. Typhimurium* community by changing water physicochemical properties, including nutrient availability, cationic metal solubility, organic carbon characteristics, and electrical conductivity, which might exert a more direct influence on bacterial community structure [36]. Water pH might also directly stress and select for different aqueous bacteria taxa. Water microbes that are more sensitive to pH change might die off faster than those more tolerant to pH changes [21]. Extreme pH values may impose a significant stress to certain taxa, while others may have higher tolerance [37]. Results from this study showed that pH had a significantly positive effect on the *ttd* values. *S. Typhimurium* strain can grow at a variety of pH values [38], and the effect of pH on bacterial inactivation in aquifers is lowest at pH levels between 6 and 8 [39], and greatest in acid conditions [40]. Overall, our conclusion that pH may affect *S. Typhimurium* is largely in agreement with some survival results reported by others [28,41]. However, it should be noted that the pH range in our current study was not large enough to cover extreme cases, and such results should be interpreted with caution.

In the current study, it was found that the first log reduction time (*δ*) was significantly (*p* < 0.05) correlated with *ttd*s. This is well in line with our previous report showing that *δ* was positively related with *ttd* of *E. coli* O157:H7 in well waters, as well as in agricultural soils [15,41]. Since *δ* is easier to be obtained, it could be an alternative indicator of the *E. coli* strains that survived in soils. Additionally, when different pathogenic bacterial strains were taken into account, the *ttd*s were found to be positively correlated with the shape parameter (*p*), suggesting that a concave (*p* < 1) survival shape may correspond to shorter *ttd*, while a convex (*p* > 1) survival shape may correspond to a longer *ttd* [41].

In this study, the measured water physicochemical properties could explain 60% of the overall variation of survival behavior (*ttd*, *δ*, and *p*), leaving nearly 40% unexplained. This could be due to the unmeasured environmental variables, as discussed above. *S. Typhimurium* might have a complex interaction with microbial communities of the well waters. The potential interaction mode could be predation, substrate competition, and antagonism. It was accepted that an aqueous ecosystem with a reduced diversity index might favor the survival of pathogens spiked into the samples [42], while an aqueous ecosystem with a higher biodiversity index might be more immune to external bacterial invasion. Since the water samples used in the current study were not filtered through 0.45 or 0.22 μm filters, the bacteria that existed in the water samples may at least compete for nutrients (e.g., carbon, nitrogen, and trace element) with pathogens introduced into the samples [30]. The overall survival of *S. Typhimurium* in well water samples might be a function of a combination of water samples’ physical, chemical, and biological factors, as observed in soil samples [37]. Obviously, further investigation was required to elucidate the correlation between *S. Typhimurium* survival behavior and the composition and structure of indigenous bacterial, fungal, viral, and protist communities in well waters. Additionally, a survey of the total population, total number of domestic animals, and identification of the super-shedders of *S. Typhimurium* in the sampling area would be of great value to explain the overall well water quality and the survival behavior of *S. Typhimurium* in well water samples.

It should be noted that in our study, only culturable *S. Typhimurium* cells were counted, while viable but non-culturable (VBNC) cells were not counted. For VBNC cells, other techniques, such as real time quantitative-polymerase chain reactions (RT-qPCR) and flow cytometry could be applied. The survival model only described the cells above the detection limit, while for the cells below the detection limit, the model might not be valid.

In summary, we investigated the survival profiles of *S. Typhimurium* in well water collected from a rural area located in southern Changchun, China. Overall, most of the survival curves displayed a concave shape, and *S. Typhimurium* could survive for up to 80 days. Further analysis revealed that the survival time (*ttd*) was significantly influenced by NH_4_^+^–N and pH of the well waters. One major feature of the current study was that we not only investigated both the major factors controlling *ttd*, but also tried to probe the major well water parameters shaping the overall survival behavior (*ttd*, *δ*, and *p*) of *S. Typhimurium* in well water samples. The relatively long survival of *S. Typhimurium* highlights the potential health risks associated with this pathogen, and the need to take appropriate actions to prevent such risks.

## Figures and Tables

**Table 1:** Properties of well waters.

Sample ID	Village	Depth (m)	Temp (°C)	pH	EC (S/m)	TOC (mg/L)	TN (mg/L)	NH_4_^+^–N (mg/L)	NO_3_^−^–N (mg/L)	TP (mg/L)	Fe (mg/L)
s1	Xinlicheng	15	7.2	6.97	22.4	3.61	20.2	0.300	6.59	0.137	0.015
s2	Changshan	50	7.8	7.36	45.5	1.35	25.4	0.357	4.16	0.123	0.025
s3	Xinnong	20	7.5	7.20	125	2.60	62.8	0.150	54.5	0.042	0.022
s4	Liushu	40	7.5	7.32	20.6	1.10	21.0	0.101	1.05	0.023	0.258
s5	Yanjia	40	8.4	7.28	28.6	0.681	23.8	0.212	8.37	0.049	0.021
s6	Xinxingxiang	40	8.9	6.38	29.3	0.120	17.0	0.329	0.483	0.012	0.089
s7	Yushu	34	7.9	6.60	20.1	0.715	17.2	0.254	2.52	0.056	1.575
s8	Xintun	20	8.1	6.82	50.7	1.80	7.29	0.158	0.800	0.002	0.027
s9	Yueshan	15	7.9	7.38	82.2	2.69	49.1	0.696	19.8	0.028	0.017
s10	Lvhua	5	5.5	7.40	52.0	1.38	29.4	0.367	27.9	0.026	0.016
s11	Nonglin	7	5.4	6.53	69.8	0.363	21.1	0.290	19.5	0.007	0.038
s12	Yongjiu	10	7.2	7.12	12.3	2.34	62.2	0.228	59.2	0.023	0.022
s13	Changlingzi	60	7.8	7.23	92.0	2.19	34.4	0.422	27.2	0.004	ND
s14	Pingan	60	7.7	6.95	19.6	0.382	12.2	0.282	10.6	0.011	0.003
s15	Yihe	50	7.4	6.82	18.3	0.001	20.2	0.212	9.24	0.009	0.066

EC, electrical conductivity; TOC, total organic carbon; TN, total soluble nitrogen; TP, total soluble phosphorus; Fe, total soluble iron. ND, not detected. The upper limits of Chinese Standards for Drinking Water Quality (GB5749-2006) for NH_4_^+^–N, NO_3_^−^–N and Fe are 0.5, 20 and 0.3 mg/L, respectively, and the standard for pH is between 6.5 and 8.5.

**Table 2:** Stepwise multi-regression analysis of water properties and survival time (*ttd*) of *S. Typhimurium* in well water samples.

Regression Equation	*R* ^2^	*F* Value	*T* Value and Partial Correlation Coefficients (*r*)		
				*T* Value	*r*
*ttd* = −114.4(±66.4) + 19.4(±9.6) × pH + 66.9(±22.4) × NH_4_^+^–N	0.580	8.280 **	pH	2.017 **·**	0.387
			NH_4_^+^–N	2.988 *	0.574

*ttd*, time to reach detection limit (day); **·**, * and ** denotes statistical significance at the 0.1, 0.05, and 0.01 level, respectively.
